# Bortezomib administered prior to temozolomide depletes MGMT, chemosensitizes glioblastoma with unmethylated *MGMT* promoter and prolongs animal survival

**DOI:** 10.1038/s41416-019-0551-1

**Published:** 2019-08-15

**Authors:** Mohummad Aminur Rahman, Andrea Gras Navarro, Jorunn Brekke, Agnete Engelsen, Christian Bindesbøll, Shahin Sarowar, Marzieh Bahador, Ersilia Bifulco, Dorota Goplen, Andreas Waha, Stein Atle Lie, Bjørn Tore Gjertsen, Frode Selheim, Per Øyvind Enger, Anne Simonsen, Martha Chekenya

**Affiliations:** 1 University of Bergen, Department of Biomedicine, Bergen, Norway; 2 Haukeland University Hospital, Department of Oncology, Bergen, Norway; 3 University of Oslo, Department of Molecular Medicine, Institute of Basic Medical Sciences and Centre for Cancer Cell Reprogramming, Institute of Clinical Medicine, Oslo, Norway; 4 University of Bergen, Department of Clinical Science and Core Facility for Metabolomics, Bergen, Norway; 5 University of Bonn, Department of Neuropathology, Bonn, Germany; 6 University of Bergen, Department of Clinical Dentistry, Bergen, Norway; 7 University of Bergen, Department of Clinical Science, Bergen, Norway; 8 Haukeland University Hospital, Department of Internal Medicine, Hematology Section, Bergen, Norway

**Keywords:** Targeted therapies, CNS cancer

## Abstract

**Background:**

Resistance to temozolomide (TMZ) is due in part to enhanced DNA repair mediated by high expression of O^6^-methyl guanine DNA methyltransferase (*MGMT*) that is often characterised by unmethylated promoter. Here, we investigated pre-treatment of glioblastoma (GBM) cells with the 26S-proteasome inhibitor bortezomib (BTZ) as a strategy to interfere with *MGMT* expression and thus sensitise them to TMZ.

**Methods:**

Cell lines and patient GBM-derived cells were examined in vitro, and the latter also implanted orthotopically into NOD-SCID C.B.-Igh-1b/lcrTac-Prkdc mice to assess efficacy and tolerability of BTZ and TMZ combination therapy. *MGMT* promoter methylation was determined using pyrosequencing and PCR, protein signalling utilised western blotting while drug biodistribution was examined by LC-MS/MS. Statistical analysis utilised Analysis of variance and the Kaplan–Meier method.

**Results:**

Pre-treatment with BTZ prior to temozolomide killed chemoresistant GBM cells with unmethylated *MGMT* promoter through MGMT mRNA and protein depletion in vitro without affecting methylation. Chymotryptic activity was abolished, processing of NFkB/p65 to activated forms was reduced and corresponded with low MGMT levels. BTZ crossed the blood–brain barrier, diminished proteasome activity and significantly prolonged animal survival.

**Conclusion:**

BTZ chemosensitized resistant GBM cells, and the schedule may be amenable for temozolomide refractory patients with unmethylated *MGMT* promoter.

## Background

Glioblastoma (GBM) is the most frequent and lethal primary brain malignancy in adults. Standard therapy, including surgery and temozolomide (TMZ), administered concomitantly with fractionated ionising radiotherapy extends median survival to only 14.6 months.^1^ Thus, an urgent, unmet need for strategies that can overcome chemoresistance grows as society ages, and GBM-reported incidence and/or diagnosis increases.

TMZ is an alkylating chemotherapy that generates toxic O^6-^methylguanine DNA adducts. During replication, these adducts become irreversibly bound by the suicide DNA repair enzyme, O^6^-methyl guanine (O^6^-MeG) DNA methyltransferase (MGMT), which is then ubiquitinated and targeted for degradation.^2^ MGMT-mediated DNA repair is a major cause of treatment failure,^3,4^ and thus promoter methylation status in patient tumours has become a predictive biomarker for response to TMZ. The median survival of patients with methylated *MGMT* versus unmethylated promoter in tumours was 21.7 versus 12.7 months after TMZ chemoradiation.^4^

Transcriptional regulation of *MGMT* expression is complex, and several factors, including SP-1, AP-1, CEBP, HIF-1α and NFκB, have been implicated. NFκB signals downstream of PI3K/AKT signalling, which is constitutively activated in many GBMs due to mutations in the tumour suppressor *PTEN.*^5,6^ NFκB binds the *MGMT* promoter at two sites and regulates its expression via p65 (RelA).^7^ Activation of NFκB is dependent on proteasomal activity, which catalyses the proteolysis of an inactive precursor into transcriptionally active p50/p65 subunits, as well as the destruction of the inhibitory factor IκBα.^8,9^ Thereafter, p65 translocates to the nucleus and *MGMT* is transcribed. Targeting the proteasomal pathway might thus diminish NFκB transcription-dependent *MGMT* expression through retention of the inactive complexes in the cytoplasm.^10^

Bortezomib (BTZ, Velcade) is a proteasome inhibitor that reversibly blocks the chymotryptic-like activity of the β5 and β1 subunits of the 20S proteasome. The drug is approved for the treatment of multiple myeloma and mantle cell lymphoma,^11^ and is an attractive candidate for other malignancies. For GBM, BTZ has been shown to reduce levels of MGMT in T98G cells in vitro, which harbour a partially unmethylated *MGMT* promoter and further shown to induce apoptosis in these cells when administered prior to TMZ^12,13^ associated with activation of NFκB, MAPK, STAT3 and HIF-1α pathways. However, these studies were performed using homogeneous cell lines (U87 and T98G) in short-term cell viability assays after 24 h with considerably high doses (>100 nM) of bortezomib.^12–14^ In investigating the involvement of the NFkB/p65 pathway, these studies did not use phosphor-site-specific antibodies, challenging interpretation of activation of the pathway. Neither preclinical studies in vivo on heterogeneous patient-derived GBM cells with an unmethylated *MGMT* promoter nor indeed clinical trials have investigated BTZ in sensitisation schedule for Temozolomide, as we propose in this paper. Previous preclinical studies administered BTZ as monotherapy in tumours implanted at ectopic sites.^15,16^ Several clinical studies investigated BTZ as an add-on therapy to low doses of TMZ^17^ combined with radiotherapy,^18^ bevacizumab,^17^ Tamoxifen^19^ or HDAC inhibitors,^20^ or as monotherapy^21^ and all deemed it safe and tolerated.^19,21,22^ A recent phase II study reported some survival benefit^23^ after prolonged treatment.

Depletion of MGMT, using the artificial substrate O^6-^benzylguanine, has been shown to sensitise tumour cells to alkylating chemotherapy.^24^ We thus, hypothesised that BTZ pre-treatment may sensitise chemoresistant GBM cells to TMZ. We used several patient-derived tumour cells in vitro and in vivo and treated the animals with human equivalent doses of both drugs for added clinical relevance. NOD-SCID mice permit engraftment of primary cells and the resultant patient-derived GBM xenografts at orthotopic sites demonstrate similar biological characteristics, molecular genetics and therapy responses of the patient-derived parental tumour.^25,26^ We tested the in vivo efficacy of pre-treatment with BTZ followed by TMZ in resistant and sensitive patient-derived tumours in vivo. We investigated responses to two human equivalent TMZ doses and could demonstrate that 164 mg/m^2^ that was closest to the 200 mg/m^2^ administered to recurrent patients was most effective as it (i) prolonged progression free survival and (ii) overall survival in animals bearing GBM with unmethylated *MGMT* promoter. We also demonstrated that BTZ administered 48 h prior to this effective TMZ dose crosses the blood–brain barrier (BBB), depleted *MGMT* mRNA levels and attenuated proteasome activity in vivo, thus accounting for restored TMZ sensitivity and prolonged animal survival. Booster BTZ+TMZ combinations during follow-up after first MRI evidence of relapse may further extend overall survival in the BTZ+TMZ164 mg/m^2^ treatment group. Our preclinical study is the first in vivo study investigating efficacy of a sequential, sensitisation treatment schedule with Bortezomib and Temozolomide. Our extensive characterisation of potential toxicity through analyses of liver function, bone marrow-derived cells, coagulation profiles and animal weights proved the treatment safe and tolerated, consistent with patient clinical trials.^19,21–23^ Our results suggest that such a sensitisation schedule may be of clinical benefit for GBM patients with unmethylated *MGMT* promoter.

## Methods

A detailed description of the methods used in the present study can be found in Supplementary Information.

### Ethics statement

Approval for the collection of primary human tumour samples into a centralised biobank (REK Vest 013.09/20879) with patients´ written informed consent and approval for use in experimental procedures in the project (REK2018/71) was obtained from the REK Vest regional ethical committee and the Norwegian Data Protection Agency. The animal study protocols were approved by The Norwegian Animal Research Authority (Oslo, Norway, FOTS ID 9460) in accordance with the European Convention for the Protection of Vertebrates Used for Scientific Purposes [Scientific Procedures] Act 1986, as well as the ARRIVE guidelines.^27^

### Isolation of primary glioma cells

P3, 2012–018 and BG7 patient-derived GBM cells were expanded from biopsies obtained from patients undergoing tumour resection at the neurosurgery department (Haukeland University Hospital; Bergen, Norway). These cells, as well as long-term cell lines (U87, T98G, HF66 and A172), were propagated as monolayers in Dulbecco’s modified eagle medium (DMEM, Sigma-Aldrich; St. Louis, MO, USA) supplemented with 10% foetal bovine serum, non-essential amino acids, 100 U/mL penicillin/streptomycin and 400 μM L-glutamine (complete medium; all Cambrex; East Rutherford, NJ, USA) at 37 °C in a humidified atmosphere of 5% CO_2_. Patient-derived cells and cell lines were authenticated by short tandem repeat loci (STR) analysis as described in Supplementary Methods. Profiles from patient-derived cells were compared with those from the original parental biopsy (P3, 2012–018 and BG7), and cell lines were authenticated against the profiles from previously fingerprinted stocks or manufacturer provided profiles, e.g., ATCC repository (T98G, A172 and U87) or manufacturer (NHA) Applied Biological Materials Inc. (Richmond, BC, Canada).

### Drugs

Temozolomide (TMZ, 2706/50, Tocris Bioscience; Bristol, UK) and MG-132 (M7449, Sigma-Aldrich) were dissolved in DMSO and stored at –20 °C. Bortezomib (BTZ, Velcade®, 576415, Janssen; Lysaker, Norway) and Carfilzomib (PR-171; Haukeland University Hospital pharmacy, Bergen, Norway) were dissolved in 0.9% v/v sodium chloride and stored at –80 °C and –20 °C, respectively. The human equivalent doses (HED) were calculated as previously reported;^28^ mouse 25 mg/kg and 50 mg/kg TMZ doses are equivalent to human doses of 84 mg/m^2^ and 164 mg/m^2^, respectively. We used BTZ 0.5 mg/kg in vivo, which is equivalent to a human dose of 1.3 mg/m^2^.

### Clonogenic survival assay

Cells were seeded at plating efficiency (1000 cells/well) in six-well plates and exposed to TMZ (72 h: 12.5–250 μM) or BTZ (48 h: 5–25 nM) as monotherapy or in combination. After the treatment period, culture medium was replaced with fresh medium followed by further observation for 14 days. After 14 days, colonies were fixed, stained in 6% glutaraldehyde/0.5% Crystal violet and counted as previously described.^29^ Surviving fractions were calculated from experiments performed in triplicate, and data represents at least three independent experiments.

### Intracranial implantation of glioblastoma spheroids

P3 and BG7 GBM cells were propagated in vitro in the Neural Basal (NB) medium (Invitrogen, Hämeenlinna, Finland) supplemented with 1% GlutaMAX (Invitrogen, Carlsbad, CA, USA), 2% B-27 (Invitrogen), 5% penicillin–streptomycin, 10 mg/ml epidermal growth factor and fibroblast growth factor (PeproTech SE, Stockholm, Sweden) at 37 °C in a humidified atmosphere of 5% CO_2_. For in vivo experiments, standardised neurospheres containing 10^4^ cells/sphere were established by centrifuging cells in a CorningTM Clear Polystyrene V shaped-bottom 96-well plates in 0.5% v/v methylcellulose-NB for 2 h at 2250 rpm and 33 °C. Spheroids were cultured for 1 week in the incubator at 37 °C and 5% CO_2_, and media was changed at day 4. P3 and BG7 tumour spheroids (five per animal) were implanted intracranially into male and female 7-week-old (20 g) severe combined immunodeficient (NOD-SCID) mice (C.B.-Igh-1b/lcrTac-Prkdc) (Janvier Labs; Le Genest-Saint-Isle, France) after anaesthetic with Servoflurane (Abbott Laboratories Ltd., Maidenhead, UK) as specified in Supplementary Information and previously described.^30^ After surgery, animals were allowed to recover from anaesthesia in a pre-warmed incubator and administered Temgesic (0.05 mg/kg, intraperitoneal injection, Indivior UK limited, Berkshire, UK) as analgesia for 3 days. The treatment schedule is illustrated in Fig. 3a. Both sexes were used in order to rule out hormonal effects on tumour growth and reduce pressure on animal breeding for experimental purposes. The animals were housed in pairs in internally ventilated, closed cages (IVC) with standard bedding (NESTPAK™, Datesand, Manchester, UK). The animals were kept in an isolation facility at 25 °C (55% relative humidity) on a standard 12 h night and day cycle in a specific pathogen-free environment, and fed a standard pellet chow and provided tap water ad libitum. Animal husbandry protocols were followed where animals were monitored daily according to humane endpoint guidelines by experimental staff and independently, by animal husbandry staff, including inhouse veterinarian. Mice were sacrificed by CO_2_ inhalation and decapitation when neurological symptoms of rotational behaviour, reduced activity, grooming and or upon 20% body weight loss. Further information can be found in Supplementary Material.

For calculation of sample sizes, we used the formula: *n* = ((2 × (s)^2^)/(gr1 − gr2)^2^) × F(a, b)) (http://www.stat.ubc.ca/~rollin/stats/ssize/n2.html), where Gr1 = 9 represents the average survival in weeks for control animals with the same tumour type; Gr2 = 10.6, minimum average survival in weeks, which we believe is necessary for the survival to be of clinical relevance, sigma (s) = 1.0, standard deviation of the average survival of control animals with the same tumour type. A two-sided *T* test was chosen, where a = *p* < 0.05 significance level, b = 0.80, power for this experiment. With this power calculation, we determined that *n* = 7 animals per group were required to show a significant difference of at least 1.4 weeks (10 days) between the control group and the treatment group.

### LC-MS-MS assay for detection of bortezomib in mouse brain tissue

In all, 250 µl of acetonitrile (Honeywell, Seelze, Germany), containing bortezomib-d8 (Toronto Research Chemicals, Toronto, Canada) as an internal standard, and 250 µl of a 1% aqueous solution of formic acid (Sigma-Aldrich, Steinheim, Germany) were added to cryopreserved mouse brain tissue. The samples were then homogenised in a Tissuelyser ball mill from Qiagen (Crawley, UK), thereafter sonicated for 5 min and centrifuged for 10 min at 2 °C. In total, 100 µl of the supernatant was removed and evaporated to dryness under flowing nitrogen. In all, 100 µl of a 1% *v/v* formic acid was used for reconstitution, and the samples were then subjected to liquid–liquid extraction by adding 700 µL of methyl tert-butyl ether (Sigma-Aldrich, Steinheim, Germany). Overall, 600 µl of the supernatant was evaporated to dryness and reconstituted in 100 µl of acetonitrile: 0.01% formic acid (50:50, *v/v*) and analysed on an Acquity UPLC system connected to a Xevo TQ-S tandem mass spectrometer (Waters, Milford, MA, USA). The compounds were separated on a Waters BEH C18 column (50 × 2.1 mm, 1.7 -µm particle size), which was developed by gradient elution over 14 min using water:acetonitrile (9:1, *v/v*) as a weak mobile phase and acetonitrile as a strong mobile phase. Bortezomib was detected in the positive mode by using the 367.1 > 226.0 transition as quantifier, and the 367.1 > 207.9 as qualifier. The ratio between quantifier and qualifier was calculated to check the purity of the chromatographic peaks. Similarly, the 375.2 > 233.9 and 375.2 > 215.1 transitions were used for bortezomib-d8. Brain tissue from untreated mice was used for calibration after adding known amounts of bortezomib standard (Toronto Research Chemicals) to the precipitation solution. Final concentrations of bortezomib were adjusted for the weight of each brain sample, which ranged from 11 mg to 36 mg. Bortezomib was stable in acetonitrile: aqueous formic acid for 48 h at room temperature (99 ± 16.8%) and during five freeze–thaw cycles (108 ± 13.3%). They were also stable for one hour after adding the precipitation solution and for 24 h in the autosampler (95 ± 10.8%).

### Statistical analysis

Survival outcome of tumour-bearing mice was analysed using the Kaplan–Meier method with the Mantel–Cox log-rank test.^31^ One-way ANOVA and two-way ANOVA were used to analyse data with one dependent variable, or two or more dependent variables, compared in two or more groups. Bonferroni or Tukey’s post hoc correction for multiple testing was used. Descriptive statistics were reported as the mean ± standard error of the mean (S.E.M.) unless otherwise stated. Two-sided *P*-values < 0.05 were considered significant (shown as *P < 0.05, **P < 0.01, ***P < 0.001, and ****P < 0.0001). All graphs represent the mean ± S.E.M. of at least three independent experiments. All statistical analyses were performed in Stata version 13.1 (College Station, TX, USA) or GraphPad Prism v6.07 (La Jolla, CA, USA).

## Results

### GBM cells are most sensitive to BTZ

**Fig. 1:**
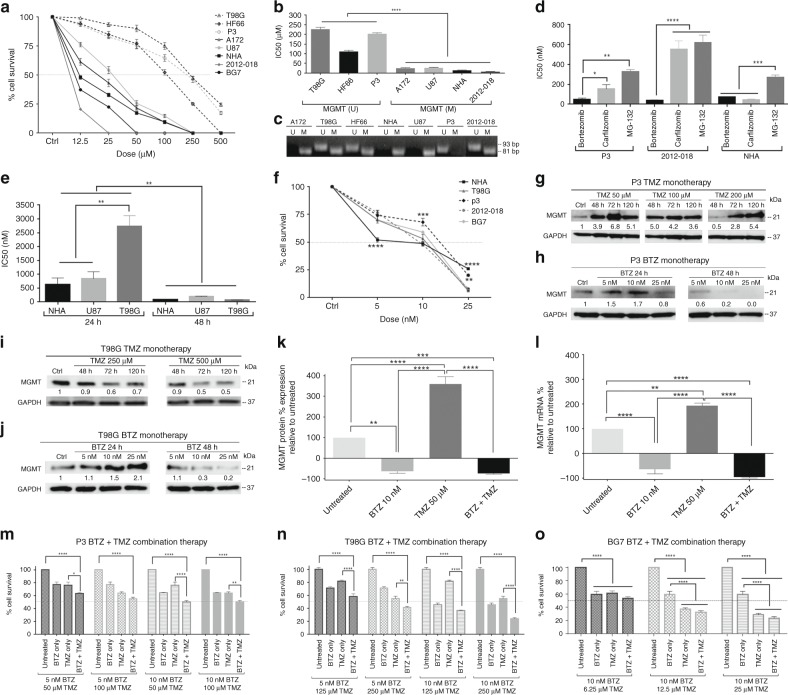
GBM cells are sensitive to BTZ due to depletion of MGMT protein and mRNA. (**a**) Mean % ± standard error of the mean (S.E.M.) survival of control NHA, glioma cell lines and patient-derived cells treated with TMZ for 72 h using clonogenic assays. (**b**) Mean ± S.E.M. clonogenic survival on IC_50_ doses (µM) of TMZ in *MGMT* methylated (M) or unmethylated (U) GBM cells. (**c**) Ethidium bromide-stained agarose gel showing amplified DNA fragments corresponding to *MGMT* promoter methylation status in NHA and glioma cells. For positive control of unmethylated *MGMT* promoter HCC1569, breast cancer cells were used (Supplementary Fig. 3). MW: 93 bp, unmethylated; and 81 bp, methylated. (**d**) Mean ± S.E.M. viability on IC_50_ doses (nM) of BTZ, carfilzomib or MG-132 after 48 h treatment of P3, 2012-018 and NHA. (**e**) Mean ± S.E.M. viability on IC_50_ doses (nM) of BTZ after 24 or 48 h treatment of NHA, U87 and T98G. (**f**) Mean % ± S.E.M. survival of tumour cells treated with BTZ for 48 h using clonogenic assays. Western blots of MGMT protein levels after TMZ dose and time for (**g**) P3, (**i**) T98G cells, and after BTZ treatment for (**h**) P3, (**j**) T98G, 24 and 48 h. % relative to control of MGMT (**k**) protein and (**l**) mRNA expression from P3 GBM cells after treatment. Clonogenic surviving fractions of (**m**) P3, (**n**) T98G and (**o**) BG7 cells after treatment. Each experiment was performed in triplicate, and the data represent the mean ± S.E.M. of at least three independent experiments, *P < 0.05, **P < 0.01, ***P < 0.001 and ****P < 0.0001

We first investigated the responses of patient-derived GBM primary cells and cell lines of different genetic backgrounds (Supplementary Table I) to monotherapy with the alkylating agent TMZ. We began by confirming the tumour cells´ *bonafide* identity by short tandem repeat loci (Supplementary Information). P3, T98G and HF66 were the most TMZ-resistant GBM cell lines, while A172, BG7, 2012–018, U87 and control normal human astrocytes (NHA) were the most sensitive (Fig. 1a, b). TMZ sensitivity was consistent with the methylation status of the *MGMT* promoter (*P* < 0.0001, Fig. 1a–c; Supplementary Table I). We then investigated the cytotoxic efficacy of BTZ compared with other proteasome inhibitors carfilzomib and MG-132 in these same cell lines (Supplementary Table I; Fig. 1d). P3 cells were more sensitive to BTZ compared with carfilzomib (*P* < 0.05) or MG-132 (*P* < 0.01), likewise 2012–018 cells for both drugs (*P* < 0.001) where IC_50_ doses for BTZ at 48 h were significantly lower than for carfilzomib or MG-132 (Fig. 1d; Supplementary Table I). Long-term cell lines were also most responsive to BTZ after 48 h (*P* < 0.01, Fig. 1e; Supplementary Table I). However, clonogenic survival analysis following BTZ treatment revealed 10 nM (range 8–12 nM) as the physiologically relevant IC_50_ dose in the cell types tested (Fig. 1f; Supplementary Table I).

### BTZ sensitises GBM cells to TMZ chemotherapy through depletion of MGMT

MGMT protein levels were upregulated with increased TMZ dose and treatment duration by fourfold to sixfold in P3 cells where the *MGMT* promoter was unmethylated (*P* < 0.0001, Fig. 1g, k). Protein levels were only gradually reduced with a protracted treatment period of 72–120 h in T98G cells with a partially unmethylated *MGMT* promoter (Fig. 1i). In contrast, BTZ monotherapy at 10 nM efficiently suppressed MGMT protein and mRNA levels by 80 ± 18.8% in P3 cells (Fig. 1h, k, l, *P* < 0.01 and *P* < 0.0001, respectively), and decreased MGMT protein by 70% in T98G (Fig. 1j) after 48 h. However, treatment with low dose BTZ for 24 h did not lead to decreased MGMT protein expression in P3 or T98G cells (Fig. 1h, j). In contrast to TMZ, cells were sensitive to BTZ treatment, which led to reduced levels of MGMT protein after 48 h, but not 24 h, indicating that this time point might be optimal for combination treatment.

**Fig. 2:**
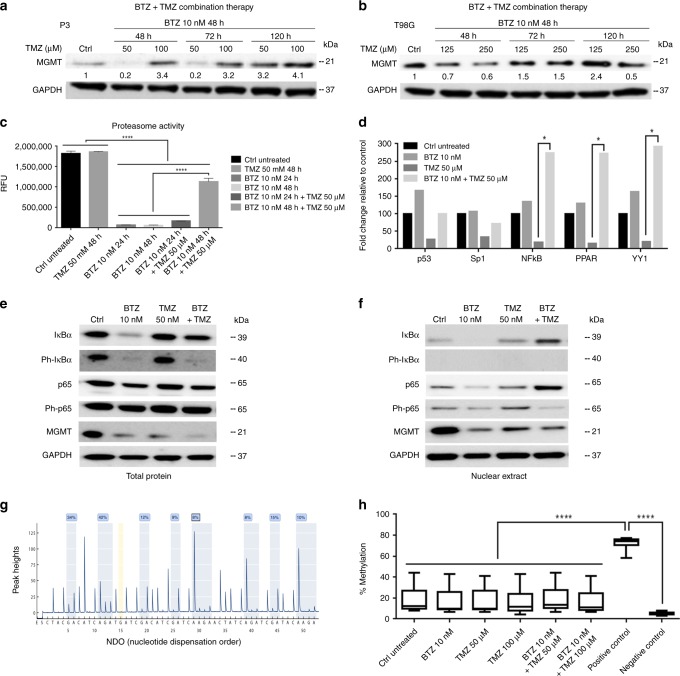
Decreased NFκB transcription is associated with reduced MGMT protein levels. MGMT protein levels after combination BTZ+TMZ treatment in (**a**) P3 and (**b**) T98G cell lines. GAPDH was used as a loading control. Band intensity expressed as a ratio after normalisation to GAPDH. (**c**) Chymotryptic activity of the β1 and β5 subunits measured as relative fluorescence units (RFU/380/460 nm) of fluorophore 7-amino-4-methylcoumarin in cells treated as indicated. (**d**) Fold change in transcription factor activity in nuclear extracts from P3 cells. NFκB/p65 subunit levels in P3 cell extracts from (**e**) total protein, and (**f**) nuclear fraction after treatment, as indicated. (**g**) Representative peak heights and nucleotide disposition order (NDO) within *MGMT* promoter CpG region (**h**) % *MGMT* promoter methylation in P3 cells after treatment. Each experiment was performed in triplicate, and the data represent the mean ± S.E.M. of at least three independent experiments, *P < 0.05 and ****P < 0.0001

Because MGMT mRNA and protein levels were decreased under BTZ treatment, we investigated whether BTZ pre-treatment might sensitise GBM cells to TMZ when MGMT levels are low. Pre-treatment with 10 nM BTZ for 48 h followed by TMZ treatment significantly reduced the survival fraction of P3 and T98G GBM cells (unmethylated *MGMT* promoter) compared with TMZ monotherapy (*P* < 0.01 to *P* < 0.0001, Fig. 1m, n, respectively) and untreated controls (all *P* < 0.0001), but not BTZ alone (*P* > 0.05, Fig. 1m, n). In contrast, BG7 patient-derived GBM cells (methylated *MGMT* promoter) were highly sensitive to either TMZ or BTZ (*P* < 0.0001, Fig. 1o), and were thus not further sensitised under combination treatment. These results provided proof-of-concept for our selected focus of combination treatment on resistant tumours with unmethylated *MGMT* promoter. The IC_50_ dose of TMZ in combination with BTZ in P3 and T98G cells was reduced to half of the IC_50_ dose of either drug in monotherapy (Fig. 1m, n). MGMT protein and mRNA levels were also decreased in P3 under combined treatment compared with TMZ monotherapy (Fig. 1k, l and Fig. 2a, b). Combination treatment also reduced MGMT protein by 30–40% in T98G (Fig. 2b), indicating that low dose BTZ synergised with TMZ in killing resistant cells, potentially through suppression of MGMT mRNA and protein expression.

### Decreased nuclear NFkB/p65 transcription factor is coincident with MGMT protein loss

To elucidate potential mechanisms for the loss of MGMT mRNA and protein in the presence of BTZ, we first confirmed that BTZ blocked the chymotryptic activity of the 20S proteasome (Fig. 2c). Chymotryptic activity remained unchanged at 48 h in TMZ-treated P3 cells relative to controls. In contrast, the catalytic activity of β5 and β1 subunits was significantly inhibited whether cells were treated with BTZ alone for 24 and 48 h or combined with TMZ (*P* < 0.0001, Fig. 2c). However, more than half of the catalytic activity was restored after the period spanning combination BTZ for 48 h and TMZ treatment for further 48 h.

We then assessed protein levels of transcription factors under proteasome blockade using an activated transcription factor protein array. NFκB, as well as peroxisome proliferator-activated receptor (PPAR) and Ying Yang-1 (YY1), accumulated in response to BTZ alone or in combination with TMZ (Fig. 2d). Further examination of NFκB subunits using total and nuclear protein extracts indicated that although phosphorylated p65 remained unchanged in total protein lysates under all treatments relative to controls, BTZ led to reduced levels of phosphorylated/activated p65 in the nucleus (Fig. 2e, f). Total and nuclear levels of MGMT protein were also reduced in the presence of BTZ, indicating a potential decrease in p65-mediated transcription of the gene (Fig. 2d–f). Pyrosequencing of the *MGMT* promoter confirmed that only a few CpG islands were methylated in the *MGMT* promoter of P3 cells, which did not change after treatment with either BTZ alone or combination BTZ+TMZ treatment (Fig. 2g, h).

### Combination treatment rapidly shrinks tumour growth and prolongs survival of mice

**Fig. 3:**
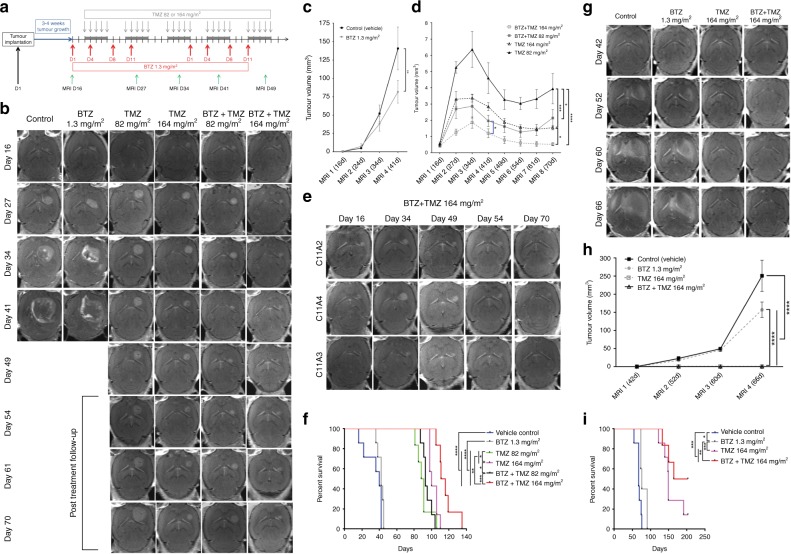
Combination BTZ+TMZ 164 mg/m^2^ treatment suppresses tumour growth and prolongs survival. (**a**) Schematic outline showing tumour implantation, treatment schedule and frequency of MRI monitoring. (**b**) Axial contrast enhanced T1-weighted longitudinal MRI follow-up of the same P3-bearing animal from each treatment group as indicated in days. (**c**) Mean ± S.E.M. of 3D measured tumour volumes (mm^3^) from T1-weighted with contrast images of (**c**) all P3 animals in vehicle control and BTZ 1.3 mg/m^2^ groups (Student’s *t* test, **P < 0.01), and (**d**) all animals in TMZ monotherapy or BTZ+TMZ combination as indicated. (**e**) Representative T1-weighted MRI with contrast of animals (*n* = 3) from the BTZ+TMZ 164 mg/m^2^ treatment group showing near complete tumour regression. (**f**) Kaplan–Meier curves showing percentage survival in days. (**g**) Axial contrast enhanced T1-weighted longitudinal MRI follow-up of the same BG7 tumour-bearing animal from each treatment group as indicated in days. (**h**) Mean ± SEM of BG7 tumour volumes (mm^3^) from T1-weighted as above. (**i**) Kaplan–Meier curves showing percentage survival in days. One-way ANOVA with Bonferroni correction for multiple testing, *P < 0.05, **P < 0.01, ***P < 0.001 and ****P < 0.0001

NOD-SCID mice were orthotopically implanted with GBM cells to generate xenograft models to investigate the efficacy of combination treatment in vivo. We first examined treatment in P3 xenografts with unmethylated *MGMT* promoter. The schedule for treatment is shown in Fig. 3a. Tumours treated with vehicle or BTZ monotherapy progressed rapidly and killed their hosts within 41 days post implantation (Fig. 3b, c and f). The tumours had hypointense cores and high signal, ring-enhancing edges on T1-weighted MRI with contrast that was indicative of florid tumour angiogenesis (Fig. 3b). Tumour population doubling time was significantly prolonged by 2.9 days already at day 11 post BTZ+TMZ 164 mg/m^2^ treatment compared with TMZ 164 mg/m^2^ (7.8 days vs. 4.9 days, respectively, *P* < 0.05), and by 2.3 days compared with BTZ+TMZ 82 mg/m^2^ (7.8 days vs. 5.5 days, *P* < 0.05; data not shown).

After TMZ 82 mg/m^2^ treatment was completed at day 49, tumours progressed by day 61 (Fig. 3b, d), whereas TMZ 164 mg/m^2^ treatment reduced tumour volumes slowly compared with TMZ 82 mg/m^2^ (*P* < 0.001, Fig. 3b, d). BTZ+TMZ 82 mg/m^2^ treated tumours relapsed upon discontinuation of treatment at day 49, although they were significantly smaller than tumours treated with TMZ 82 mg/m^2^ at all time points (*P* < 0.001, Fig. 3d). Tumours from animals treated with BTZ+TMZ 164 mg/m^2^ remained small throughout the treatment and follow-up (*P* < 0.0001, compared with TMZ 82 mg/m^2^, *P* < 0.05 compared with TMZ 164 mg/m^2^ and *P* < 0.05 compared with BTZ+TMZ 82 mg/m^2^, Fig. 3b, d) and were abolished entirely in 30% of cases (Fig. 3e). Thus, the combination regimen BTZ+TMZ 164 mg/m^2^ significantly prolonged survival (log-rank 39.18, df = 5, *P* < 0.0001, Fig. 3f). 16% of BTZ+TMZ 164 mg/m^2^ treated animals were alive at day 138, the termination point of the experiment, and median survival was 114 days compared with 100 days with TMZ 164 mg/m^2^ (*P* = 0.0063) or 93 days with BTZ+TMZ 82 mg/m^2^ (*P* = 0.0012, Fig. 3f).

We investigated efficacy in a second xenograft model developed with BG7 cells with methylated *MGMT* promoter. We found that animals treated with vehicle or BTZ monotherapy developed large diffusely enhancing tumours (Fig. 3g, h) that progressed rapidly and killed their hosts within 69 and 74 days, respectively (Fig. 3i). Animals treated with TMZ 164 mg/m^2^ survived a median of 149 days compared with 69 and 74 days of control and BTZ treated animals (*P* < 0.0001, respectively), while combination BTZ+TMZ 164 mg/m^2^ extended most survival of animals to 184 days (log rank _27.59_, df = 3, *P* < 0.001, Fig. 3g–i). This combination schedule thus was also of benefit in the case of tumours with methylated MGMT promoter.

### BTZ+TMZ treatment decreases tumour angiogenesis and proliferation

**Fig. 4:**
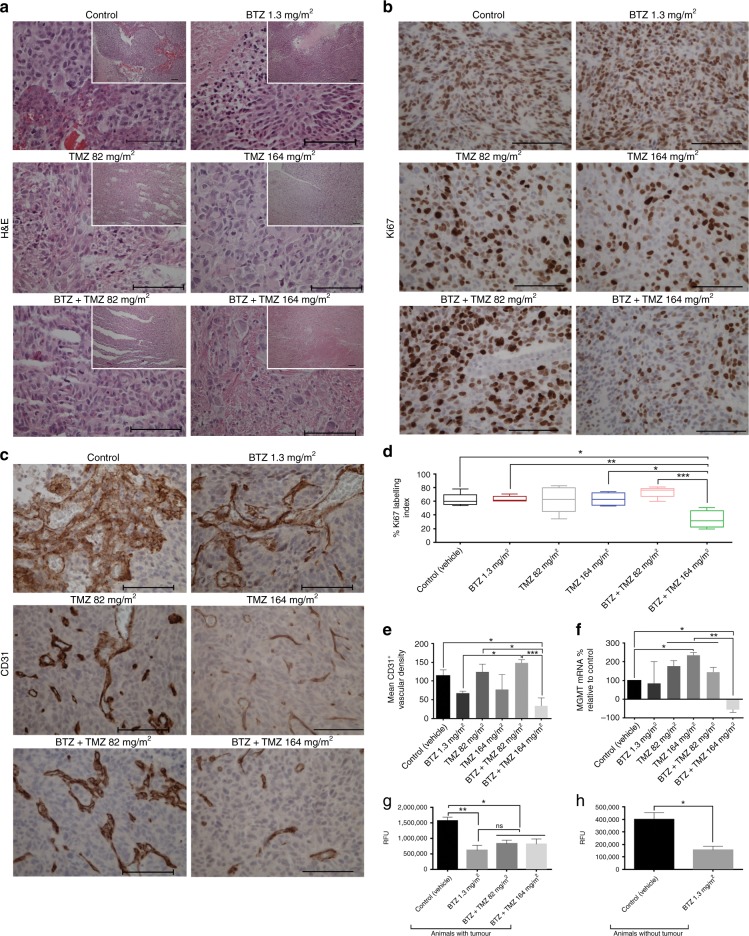
Combination BTZ+TMZ treatment reduces tumour proliferation, angiogenesis and proteasome activity. (**a**) haematoxylin and eosin (H&E) staining (insert: magnification ×200), (**b**) Ki67 staining (brown) and haematoxylin counterstain; and (**c**) CD31 staining for tumour microvessels of tumour tissue from one representative animal from each of the treatment groups indicated. Magnification ×400; scale bars, 100 μm. Percentage mean ± S.E.M. of (**d**) Ki67-labelling index and (**e**) CD31 microvascular density in all animals. (**f**) Percent *MGMT* mRNA expression in P3 GBM tumours after treatment in vivo. (**g**) Chymotryptic activity of β1 and β5 subunits of the 20S proteasome in brain tissue of tumour-bearing mice treated with BTZ alone or BTZ+TMZ combination, measured as relative fluorescence units (RFU) of fluorophore 7-amino-4-methylcoumarin (AMC) at 460 nm (*n* = 3 mice/group) (**h**) β1 and β5 chymotryptic activity in non-tumour-bearing brain tissue of untreated control or BTZ treated mice (*n* = 3 mice/group). One-way ANOVA with Bonferroni correction for multiple testing, *P < 0.05, **P < 0.01 and ***P < 0.001

Tumours treated with vehicle or BTZ monotherapy were large, highly cellular and exhibited both central and pseudopalisading necrosis with pyknotic nuclei and mitotic figures (Fig. 4a). TMZ 82 mg/m^2^ and TMZ 164 mg/m^2^ treated tumours had moderate-to-low cell density dominated by giant nucleated tumour cells, prominently fibrillary extracellular matrix and reduced mitotic figures (Fig. 4a). BTZ+TMZ 82 mg/m^2^ treated tumours were consistently large, densely cellular with pseudopalisading necrosis and pyknotic nuclei, whereas 50% (3/6) of BTZ+TMZ 164 mg/m^2^ treated tumours were small, fibrotic and necrotic with only sparsely scattered tumour cells (Fig. 4a). As degree of angiogenesis (measured by microvascular density) and ki67 proliferation index is highly correlated with tumour growth, we used these as surrogate markers that verify tumour growth kinetics in the treated animals. Tumour cell proliferation, as measured by the Ki67 labelling index, was significantly reduced after BTZ+TMZ 164 mg/m^2^ treatment compared with TMZ 164 mg/m^2^ (*P* < 0.05), BTZ+TMZ 82 mg/m^2^ (*P* < 0.001), BTZ monotherapy (*P* < 0.01) and vehicle control (*P* < 0.05, one-way ANOVA, Fig. 4b, d).

**Fig. 5:**
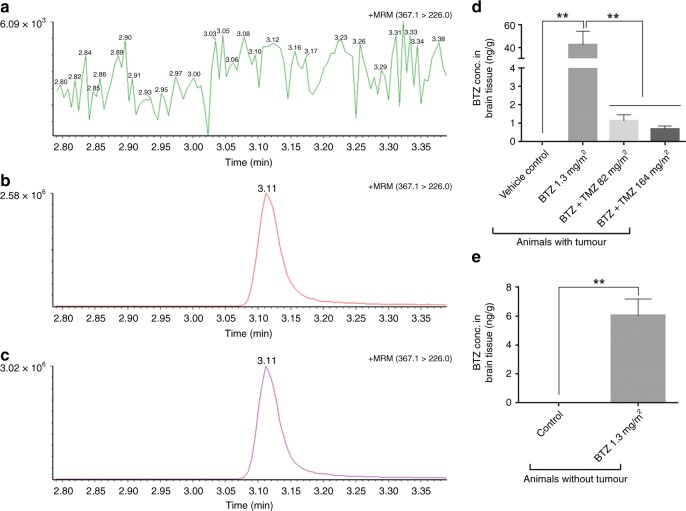
Detection of BTZ concentrations in mouse brains. Typical chromatograms in positive multiple reaction monitoring (MRM) mode for Bortezomib in (**a**) vehicle control mouse brain tissue (**b**) vehicle control mouse brain tissue spiked with the BTZ analyte. (**c**) Mouse brain tissue treated with BTZ 1.3 mg/m^2^ (on days 1, 4, 8, 11, for two cycles). Mean ± S.E.M. of BTZ tissue concentrations (ng/g) (**d**) in tumour-bearing mouse brains, and (**e**) in control or BTZ- treated mouse brains without tumour. For single agent BTZ treatment, tumour-bearing mice were killed after ~40 days, the majority within 2 days after last BTZ dose. BTZ+TMZ combination treated mice were killed after ~100 days, 45 days after last BTZ dose. Control non-tumour-bearing mice were untreated or treated on days 1, 4, 8, 11, for two cycles and killed 2 days after last BTZ dose. One-way ANOVA with Bonferroni correction for multiple testing, **P < 0.01

Tortuous microvascular proliferations were evident in control and BTZ treated tumours, in contrast to moderate microvascular density after TMZ 82 mg/m^2^ and BTZ+TMZ 82 mg/m^2^ treatment (Fig. 4c). Tumours treated with BTZ+TMZ 164 mg/m^2^ exhibited markedly reduced angiogenic vascular density, compared with BTZ+TMZ 82 mg/m^2^ (*P* < 0.001), BTZ monotherapy or vehicle control (*P* < 0.05, for both Fig. 4c, e). Expression of *MGMT* mRNA was significantly reduced, by 83% in BTZ+TMZ 164 mg/m^2^ compared with TMZ 164 mg/m^2^ monotherapy treated tumours, 65% compared with BTZ+TMZ 82 mg/m^2^ and 37% compared with TMZ 82 mg/m^2^ (*P* < 0.01 for all, Fig. 4f), indicating successful target inhibition in vivo. Furthermore, β1 and β5 chymotryptic activity was significantly reduced in BTZ-treated tumours compared with vehicle controls (*P* < 0.01, Fig. 4g) and in combination treated versus vehicle control (*P* < 0.05, Fig. 4g). The β1 and β5 chymotryptic activity was also reduced by ~25% in BTZ-treated non-tumour-bearing brain tissue compared with healthy mouse brain, (*P* < 0.05, Fig. 4h). There was no difference in chymotrytptic activity of BTZ compared with combination treated tumours. Biodistribution analyses (Fig. 5a–c) demonstrated that i.p. administered BTZ does breach the BBB and could be detected in greater concentrations in tumour tissue, and from animals killed at earlier time points (*P* < 0.01, Fig. 5d). In healthy, non-tumour-bearing mice, BTZ did cross the intact BBB, albeit with reduced concentrations (*P* < 0.01, Fig. 5e). Taken together, BTZ+TMZ 164 mg/m^2^ treatment was therefore the most effective compared with other treatment protocols, where BTZ crossed the BBB and exhibited bioactivity in both tumour and non-tumour-bearing brain tissue. It was well tolerated based on their liver function tests (alanine aminotransferases and aspartate aminotransferases); platelet counts and whole blood clotting times (Supplementary Fig. 1A–D). Measures of platelet nadirs drop during treatment but recovered rapidly to baseline (Supplementary Fig. 1A) between treatment cycles, consistent with previous reports.^32^ GBM is known to be prothrombotic rapid clotting time (Supplementary Fig. 1B). BTZ prolonged clotting time at D1 (*P* < 0.0001 compared with untreated control animals with tumour (ctrl + tumour); but already after D2, the whole blood clotting time was normalised to similar levels as in ctrl + tumour animals. As clotting factors are partially contributed by the liver, we could show that liver function (through enzymes, alanine and aspartate aminotransferase) was unimpaired (Supplementary Fig. 1C, D) respectively. LC–MS/MS characterisation of clotting profiles (Supplementary Fig. 1E) also indicated that the treatment was safe and tolerated. In addition, we measured daily the body weight of the mice treated with one cycle vs. two cycles of BTZ. There was no treatment induced severe weight loss and BTZ extended survival compared to control in BG7 tumour bearing mice (Supplementary Fig. 2A–C).  The body weight of all mice bearing P3 and BG7 tumours was also not adversely affected by the treatment (Supplementary Fig. 2D, E), confirming that the treatment was safe and tolerated.

Together, these findings further support a cooperative mechanism in which TMZ 164 mg/m^2^ and BTZ in potent salvo to promote tumour cell death by perturbing proteasome function and antioxidant regulation (Supplementary Fig. 1F, G).

## Discussion

Correlation of TMZ responsive and nonresponsive tumours to *MGMT* promoter methylation status has implicated higher levels of the enzyme in resistance to the drug. Here, we investigated a strategy for depleting MGMT levels by treatment with the proteasome inhibitor BTZ, before exposure to TMZ. We demonstrated that pre-treating with BTZ for 48 h depleted MGMT enzyme, in vitro and in unmethylated patient-derived GBM xenografts. We subsequently investigated two human equivalent TMZ doses in vivo, and demonstrated that 164 mg/m^2^ was more effective than 84 mg/m^2^ as it led to (i) rapid and sustained tumour shrinkage, and (ii) prolonged progression free and overall survival in animals bearing patient-derived GBM with unmethylated *MGMT* promoter. Finally, we found depleted *MGMT* mRNA levels in vitro and in vivo to be associated with decreased NFκB/p65  nuclear activation. The response to BTZ was in contrast to a second proteasome inhibitor, carfilzomib, which was deemed unsuitable for further investigation because it required high doses, did not kill all GBM cell types, and/or killed the normal astrocytes more efficiently, thus limiting its potential therapeutic window.

Dose intense TMZ treatment weakly attenuated expression of MGMT protein in partially unmethylated T98G,^7,33^ but not fully unmethylated P3 cells, consistent with previous findings where a TMZ dose intense regimen was deemed marginally effective.^34,35^ Several clinical studies have explored blocking MGMT to improve TMZ efficacy in treatment resistant GBM.^36–39^ Here, BTZ+TMZ 164 mg/m^2^ combination treatment extended survival of mice-bearing GBM with unmethylated MGMT promoter compared with TMZ 164 mg/m^2^ alone or a reduced dose BTZ+TMZ 82 mg/m^2^ combination treatment. BTZ+TMZ 164 mg/m^2^ treated tumours exhibited reduced *MGMT* mRNA relative to untreated controls (by 55%) and relative to TMZ 164 mg/m^2^ (by 83%), providing proof-of-concept that efficacy of the combination treatment might be coincident with MGMT inhibition.

Several transcription factors have been implicated in regulating *MGMT* expression, including NFκB.^7,40^ We demonstrated that reduced NFκB activation was associated with decreased MGMT protein and mRNA. In the canonical NF-κB pathway, p65/RelA proteins are bound and localised to the cytoplasm by IκBα. Phosphorylation of IκBα leads to its ubiquitination and proteasomal degradation, which liberates active p65/RelA complexes, allowing translocation to the nucleus and induction of gene expression. BTZ or BTZ+TMZ combination treatment blocked proteasome activity, and nuclear localisation of the phosphorylated and activated NFκB/p65 subunit became diminished, which was associated with the lowest MGMT protein levels both in the nucleus and total extracts. *MGMT* promoter methylation did not change after BTZ or BTZ+TMZ combination treatment, implicating NFκB/p65 in regulating MGMT mRNA and/or protein levels under our treatment schedule.

Although the chymotryptic-like activity of β1 and β5 subunits was effectively inhibited after 24 h by BTZ alone or in combination with TMZ, MGMT levels at this time point were not reduced. Only after treatment with BTZ for 48 h, was there a significant decrease in MGMT levels, consistent with its stability and relatively long half-life of ~60 h in promoter unmethylated cancer cells.^41^ Thus, BTZ+TMZ 50 μM combination treatment for 48 h (in vitro sensitisation dose determined to be a quarter of the IC_50_ 200 μM TMZ dose) precisely induced O^6^-MeG adducts that could not be repaired, as BTZ had effectively attenuated MGMT protein at this time point. In contrast, treatment with higher combination dose BTZ+TMZ 100 μM (half of the IC_50_ TMZ dose) generated more MGMT protein than could be depleted via proteasomal blockade, since activity of the β1 and β5 subunits was already partially restored at 48 h. MGMT levels remained lower than in control or TMZ treated cells, both in total and nuclear fractions.

The 20S proteasome activity returns to baseline within 72 h^42^ after BTZ treatment. A previous in vitro study reported BTZ administered before TMZ was more effective in T98G, but not in U87 cells where the reverse schedule was more effective.^12^ Although the authors did not allude to the reason for this, we show here that was likely due to the advantage of first depleting MGMT in the cells with unmethylated promoter, which thus sensitises them to TMZ. U87 cells harbouring methylated *MGMT* promoter respond better to TMZ and cannot be sensitised further, as we demonstrated in vivo with BG7 cells with methylated promoter. Furthermore, in our hands, 24 h was too early to see decreased MGMT protein in response to BTZ 10 nM treatment.

BTZ treatment reduced tumour volumes compared with untreated controls and marginally prolonged survival, indicating that it crossed the BBB. LC–MS/MS detected greater concentrations of the drug in the tumour-bearing compared with non-tumour brains of treated animals, indicating that the disrupted BBB^43^ contributes to improved penetrance. Furthermore, higher concentrations were detected in animals sacrificed at earlier time points. Although single agent BTZ marginally extended survival, the best effect was achieved in combination with 164 mg/m^2^ TMZ. This ability of BTZ to cross the BBB was previously demonstrated in rat brains^44^ as well as in a pharmacokinetic study of clinical trial enrolled patients where higher concentrations were measured in the GBM tumour tissue than in the corresponding plasma.^45^ Newer generation proteasome inhibitors such as Ixazomib and marizomib with improved BBB penetrance are now under evaluation in phase I and III clinical trials, respectively.

Prolonged tumour growth stasis on MRI preceded extended median survival in BTZ+TMZ 164 mg/m^2^ treated animals relative to TMZ monotherapy. Decreased tumour *MGMT* mRNA levels, as well as catalytic activity of the β5 and β1 subunits were found in tumours from these BTZ+TMZ 164 mg/m^2^ treated animals, confirming bioactivity and target inhibition at the orthotopic site. In contrast, tumours treated with combination BTZ+TMZ at a lower and less effective TMZ dose (82 mg/m^2^) expressed more endogenous antioxidant enzymes and may represent a possible mechanism of escape in vivo. This diminished efficacy of low dose TMZ may also partially explain the less effective metronomic TMZ doses investigated in clinical trials.^17^ Moreover, in some cancer types, de novo synthesis of proteasome subunits may represent a further mechanism of escape from proteasome blockade. Expression of the α4 and β1 subunits was significantly decreased in animals responsive to combination BTZ+TMZ 164 mg/m^2^. Indeed, this was confirmed by reduced chymotryptic-like activity of catalytic activity of the β5 and β1 subunits extracted from the treated tumour tissues. High expression of β1 subunit has been associated with susceptibility to lung cancer in GWAS studies and shown to promote tumour cell proliferation.^46^ Diminished levels of this subunit in the most responsive animals with prolonged tumour doubling and decreased proliferation are consistent with the improved antitumour responses.

Animals tolerated the treatments as their body weight was stably maintained throughout the treatment until they succumbed to their disease. Furthermore, measures of platelet nadirs, blood clotting time and the liver function enzymes, alanine and aspartate aminotransferase, in plasma, were consistently normalised, confirming that the treatment was safe and tolerated. Although survival extension by 16% in mice-bearing TMZ resistant, *MGMT* unmethylated GBM after BTZ+TMZ compared with TMZ alone was modest, in a recurrent setting this might amount to extended survival for this hard to treat patient group. Caution is required in interpreting findings in mouse studies to humans; however, clinical impact of this strategy will be evaluated in the ongoing clinical trial (NCT0364354) for recurrent GBM patients. Our findings in mice do realistically demonstrate the challenge of treating GBM patients.

This is the first in vivo chemosensitisation at an orthotopic site using patient-derived GBM cells with human equivalent doses of BTZ and TMZ in a combination schedule. We demonstrated that BTZ pre-treatment depleted MGMT and abrogated NFκB/p65 signalling, thus accounting for improved sensitivity to TMZ. Our sensitisation schedule may be of clinical relevance to improve survival of GBM patients with unmethylated *MGMT* promoter.

## Supplementary information

Supplementary Information

## Data Availability

The mass spectrometry proteomics data and files have been deposited to the ProteomeXchange Consortium via the PRIDE partner repository^47^ with the dataset identifier PXD007193. Western blot and other data sets used and/or analysed during this study are available from the corresponding author on reasonable request.
