# Supplementary material for: Bortezomib administered prior to temozolomide depletes MGMT, chemosensitizes glioblastoma with unmethylated MGMT promoter and prolongs animal survival
Source: Br J Cancer. 2019 Aug 15;121(7):545–55. doi: 10.1038/s41416-019-0551-1 (PMC6888814; doi:10.1038/s41416-019-0551-1)

**Supplementary Information**

**Supplementary Results**

*Treatment tolerated as coagulation factors and clotting time normalize*

We next investigated whether the combination BTZ+TMZ treatment was tolerated. As BTZ is known to induce early thrombocytopenia, we first investigated platelet counts but found no significant aberrations in BTZ treated or control animals (*P* > 0.05, One-way ANOVA, Supplementary Fig. 1A). Clotting time in control healthy mice before and after BTZ treatment was significantly shorter compared with tumour bearing mice (51 s *vs* 125 s, respectively; *P* < 0.001, Supplementary Fig. 1B). Clotting time in tumour bearing mice was further prolonged with BTZ treatment up to 1-day post treatment (125 s *vs* 200 s, respectively, *P* < 0.01), but returned to baseline for tumour bearing animals after 2 days (Supplementary Fig. 1B) indicating recovery. Blood clotting time in animals treated with both combinations, BTZ+TMZ 82mg/m^2^ and BTZ+TMZ 164 mg/m^2^ was comparable to control, tumour bearing mice (*P* > 0.05), consistent with the relatively normal platelets counts (Supplementary Fig. 1B). Together, these results indicated a temporary perturbed clotting response that was restored to baseline levels of untreated tumour bearing mice after BTZ treatment. We also examined levels of alanine and aspartate aminotransferase (ALAT and ASAT, respectively) liver enzymes in plasma as a measure of potential hepatocyte damage. ALAT levels were significantly reduced in mice treated with TMZ monotherapy, as well as combination BTZ+TMZ 164 mg/m^2^ compared with untreated controls (*P* < 0.05 for all, Supplementary Fig. 1C). ASAT levels however, remained unchanged (Supplementary Fig. 1D). Likewise, no changes in glucose or albumin levels were observed (data not shown).

*Proteasome subunits and antioxidant enzymes reduced under BTZ and TMZ treatment*

Altered regulation of the individual components of the proteasome complex should contribute to decreased proteasomal function after BTZ treatment. We thus mined the LC-MS/MS proteomics data for evidence of changes in proteins involved in the proteasome complex. The most effective treatment in prolonging survival, BTZ+TMZ 164 mg/m^2^, led to downregulation of the structural proteasome unit, proteasome α-4 subunit, compared with vehicle controls (2-fold, *P* < 0.01), BTZ monotherapy (4-fold, *P* < 0.05), and BTZ+TMZ 82 mg/m^2^ (6-fold, *P* < 0.05, Supplementary Fig. 1E and 1F). Treatment with TMZ 164 mg/m^2^ monotherapy reduced levels of the catalytic β1 subunit compared with vehicle controls (6-fold, *P* < 0.01) and BTZ+TMZ 82 mg/m^2^ (*P* < 0.05, Supplementary Fig. 1E and 1F).

Efficient clearing of oxygen radicals in tumour cells decreases efficacy of chemo- and radiation therapy. Therefore, the proteomics data was also examined for changes in enzymes involved in antioxidant metabolism that could serve as biomarkers of response. Several endogenous antioxidants including peroxiredoxin-1, -2, -6, thioredoxin and catalase were consistently downregulated in plasma of mice treated with BTZ+TMZ 164 mg/m^2^ compared with vehicle controls or BTZ monotherapy (Supplementary Fig. 1G). These proteins were also significantly reduced in mice after high dose TMZ 164 mg/m^2^ treatment alone compared with vehicle controls or BTZ monotherapy (*P* < 0.05, Supplementary Fig. 1G).

*Bortezomib monotherapy is bioactive against BG7 GBM tumour, tolerated and prolongs survival*

Monotherapy BTZ after 1 or 2 cycles reduced tumour sizes on T1-weighted MRI with contrast (Supplementary Fig. 2A). The treatment was tolerated as body weight recovered after BTZ injections, (Supplementary Fig.2B) and prolonged survival of mice bearing BG7 xenografts (Supplementary Fig.2C). The body weight of all mice bearing P3 and BG7 tumours was also not adversely affected by the treatment (Supplementary Fig. 2D and 2E), overall confirming that the treatment was safe and tolerated.

**Supplementary methods**

*Cell culture and reagents*

The human glioblastoma cell lines A172, T98G and U87 were obtained from the American Type Culture Collection (ATCC, Manassas, VA, USA) and HF66 from the Ford Cancer Center (Detroit, MI, USA). Immortalized normal human astrocytes (NHA) were obtained from Applied Biological Materials Inc. (Richmond, BC, Canada) with STR profile data. These primary human astrocytes were immortalized by transfection with human telomerase reverse transcriptase (hTERT). The established cell lines were propagated as monolayers in Dulbecco’s modified eagle medium (DMEM, Sigma-Aldrich; St. Louis, MO, USA) supplemented with 10% fetal bovine serum, non-essential amino acids, 100 U/mL penicillin/streptomycin and 400 μM L-glutamine (complete medium; all Cambrex; East Rutherford, NJ, USA) at 37°C in a humidified atmosphere of 5% CO_2_.

*Cell line authentication by STR profiling*

The cell typing authenticity reports are available from the authors upon reasonable request. Authentication of tumour cell identity was performed using highly-polymorphic short tandem repeat loci (STR) analysis against the original patient tumour or cell line DNA as template (Microsynth; Balgach, Switzerland). Briefly, tissue (~ 30 mg) was isolated from cryosectioned, frozen tumour biopsies (2012-018, BG7 and P3) and compared with corresponding tumour cell lines (2012-018, BG7 and P3). DNA from cell lines U87, A172, HF66, T98G and human normal astrocytes (NHA) was compared with previously fingerprinted stocks or manufacturer provided profiles. DNA was extracted using DNeasy Blood & Tissue Kits (Qiagen, Cat No./ID: 69506; Germantown, MD, USA). gDNA (50 µL at 50-150 ng/µL) was delivered to Microsynth for DNA fingerprinting.

*Viability assays*

For MTS viability assay, NHA, U87 and T98G cells were seeded at 5000 cells/well in a volume of 100 µL in 96-well plates. After adherence, cells were treated with 50 – 2,500 µM TMZ (Tocris Biosciences, Bristol, UK) for 72 h, and BTZ was tested within a dose range of 1.56 – 800 nM for 24 – 48 h. In the case of combination treatment, cells were exposed to 5 – 25 nM BTZ for 24 or 48 h followed by TMZ. Control groups received complete medium and DMSO vehicle. At the end of the treatment cells were rinsed with PBS and 20 µL of MTS (4,5-dimethylthiazol-2-yl)-5-(3-carboxymethoxyphenyl)-2-(4-sulfophenyl)-2H-tetrazolium) (CellTiter 96 AQueous One Solution Cell Proliferation Assay, (Promega; Madison, WI, USA) were added to each well and incubated for 4 h. Absorbance was measured at 492 nm on microplate reader. For WST-1 viability assay; NHA, P3 and 2012-018 cells were seeded at 10000 cells/well in a volume of 100 µL in 96-well plates. After adherence, Bortezomib, Carfilzomib and MG-132 were tested within a dose range of 1.56 – 800 nM for 24 – 48 h. Thereafter, 10 µL of proliferation reagent WST-1 (11644807001, Sigma-Aldrich) were added to each well and incubated for 2 h. Absorbance was read at 450 nm on the VersaMax microplate reader (Molecular Devices, Berkshire, UK). IC_50_ were calculated using Prism software version 6.07 (GraphPad; La Jolla, CA, USA). All experiments were performed in triplicate and repeated at least 3 independent times.

*Western blot analysis*

Cells were lysed in Kinexus lysis buffer: 20 mM MOPS, 5 mM EDTA, 2 mM EGTA, 30 mM NaF, 0.5% Triton X, 1 mM PMSF, pH 7.2, protease inhibitor (cocktail tablet, Roche; Basel, Switzerland), and phosphatase inhibitor (cocktail tablet, Roche). Nuclear proteins of the treated or control cells were extracted using Nuclear Extraction Kit (Signosis Inc.; Santa Clara, CA, USA). Samples (20 µg) were run on SDS/PAGE with NuPage precast 4-12% gradient gels (Invitrogen; Carlsbad, CA, USA) blots were incubated overnight at 4°C with primary antibodies (Supplementary Table II) followed by incubation 1.5h at RT with a species specific secondary HRP-conjugated antibody diluted 1:10000. Chemiluminescence detection was performed with Super Signal West Femto Maximum Sensitivity Substrate (Thermo Fisher Scientific; Bremen, Germany) on the LAS-3000 (Fujifilm Medical Systems Inc.; Stamford, Connecticut, USA). Relative protein expression levels were normalized to GAPDH and quantified using Image J software (NIH; Bethesda, MD, USA).

*Transcription factor (TF) activation profiling array*

TF activation profiling plate array (Signosis Inc.) was used to screen multiple activated TFs in nuclear extracts from BTZ, TMZ and combination treated P3 GBM cells. Nuclear proteins of treated or control cells were extracted using Nuclear Extraction Kit (Signosis Inc.) and incubated with biotin-labelled TF probe mix according to the manufacturer’s protocols. Luminescence was measured as relative light units (RLUs) on a microplate luminometer (Asys UVM 340; Marlborough, MA, USA).

*Proteasome Activity Assay*

20S Proteasome Activity Assay Kit (APT280, Millipore, MA, USA) was used to measure catalytic activity of the proteasome in cell lysates from BTZ, TMZ and combination treated P3 GBM cells following the manufacturer's instructions. Briefly, the cell lysis buffer (50 mM HEPES (pH 7.5), 5 mM EDTA, 150 mM NaCl and 1% Triton X-100) was used to extract the cell lysates. Cell lysates (10 µg) were mixed with 1X Assay Buffer. Fluorogenic peptides LLVY-AMC were mixed into the reaction to assess chymotrypsin-like activities of the 20S proteasome and incubated at 37°C for 2h. Fluorescence was measured using a plate reader (Asys UVM 340; Marlborough, MA, USA) at 355(excitation)/460(emission). Fluorophore 7-amino-4-methylcoumarin (AMC) and proteasome positive control standard curve was produced by reading a serial dilution of the reconstituted AMC Standard and proteasome positive control included in the kit.

*MGMT promoter methylation and mRNA expression*

MSP was carried out for 40 cycles using primers specific for methylated or unmethylated DNA. HCC1569 breast cancer cells were used as unmethylated control (ATCC^®^ CRL-2330). PCR products were separated on 2% agarose gels and ethidium bromide-stained bands were recorded. The primer sequences used to detect unmethylated MGMT promoter sequences were forward 5’-TTTGTGTTTTGATGTTGTTAGGTTTTTGT-3’ and reverse 5’-AACTCCACACTC TTCCAAAAACAAAACA-3’. The primer sequences used to detect methylated MGMT promoter sequences were forward 5’-TTTCGACGTTCGTAGGTTTTCGC-3’ and reverse 5’-GCACTCTTCCGAAAACGAAACG-3’. cDNA was synthesized using the iScript cDNA Synthesis kit (BIO-RAD; Hercules, California, USA) according to the manufacturer’s instructions. iQ SYBR Green from the Supermix kit (BIO-RAD) was used to detect amplified produce in the PCR reaction mixture. The reaction was run on a Roche light cycler (LC480, Roche; Indianapolis, IN, USA) for 40 cycles. The primer sequences were as follows: MGMT forward 5’- GCCGGCTCTTCACCATCCCG-3’ and reverse 5’-GCTGCAGACCACTCTGTGGCACG-3’ and internal control 18S forward 5’-CGGCTACCACATCCAAGGAA-3’ and reverse 5’-GCTGGAATTACCGCGGCT -3’. Target transcripts were normalized to 18S and analysed using the comparative *C*T (ΔΔ*C*T) method.

*Intracranial implantation of glioblastoma spheroids*

Animals were anesthetized with Sevofluran inhalation via a face mask (Abbott Laboratories Ltd., Maidenhead, UK) and placed on a stereotactic frame (Kopf Instruments; Tujunga, CA, USA). A gaseous anaesthetic was chosen over intravenous solution because inhalants permit better control over anaesthesia depth, allowing rapid animal recovery. In addition, Sevoflurane has several advantages over Isoflurane in that it allows faster induction, and recovery than isoflurane, does not irritate the airways and is better tolerated in mask inductions. A burr-hole was made with a micro-drill (Kopf Instruments, Tujunga, CA, USA) 0.5 mm posterior to the bregma and 2 mm to the right of the sagittal suture. Tumour spheres (5 per animal) were injected 2 mm below the brain surface with a Hamilton syringe. Six to seven animals were randomly assigned to each of the following treatment groups: (1) vehicle control *(n=7)*; (2) BTZ 0.5 mg/kg, human equivalent dose (HED), 1.3 mg/m^2^ *(n=7)*; TMZ monotherapy with (3) 25 mg/kg HED, 82 mg/m^2^ *(n=7)*, (4) 50 mg/kg, HED, 164mg/m^2^ *(N=7)*; combination (5) BTZ+TMZ 82 mg/m^2^ *(n=7)*, and (6) BTZ+TMZ 164 mg/m^2^ *(n=6)*. TMZ chemotherapy was administered by oral gavage 5 days/week for 5 weeks, starting at day 0 post BTZ pre-treatment. BTZ 1.3 mg/m^2^ was administered intraperitoneally on days 1, 4, 8, and 11, for two cycles with a 10-day break between cycles (Fig. 3a). The human equivalent doses were calculated according to the Federal Drug Administration´s recommendation: : <https://www.fda.gov/regulatory-information/search-fda-guidance-documents/estimating-maximum-safe-starting-dose-initial-clinical-trials-therapeutics-adult-healthy-volunteers>

The animals were weighed and monitored daily by experimental staff as well as by animal husbandry staff, including veterinarian. Disease progression was judged by common characteristics of the animal's general condition, activity, grooming, food and fluid intake, coat appearance, curving back etc. MRI was performed weekly. They were sacrificed by CO_2_ inhalation and decapitation when neurological symptoms of rotational behaviour, reduced activity, grooming, and or upon 20% weight loss. Survival time was recorded, brains were extracted and divided into two parts. One half was fixed in formalin for haematoxylin and eosin (H&E) and immunohistochemistry (IHC) staining, while the other half was cryopreserved in liquid nitrogen.

*Magnetic Resonance Imaging (MRI)*

MRI was performed with the 7 Tesla Bruker Pharmascan system (Bruker Corporation, Ettlingen, Germany) and linear volume RF coil internal diameter 23 mm. T1-weighted MR imaging was obtained using a rapid acquisition relaxation enhancement (RARE) sequence with the following parameters: RT = 900.0 ms; TE = 9.00 ms; rare factor = 4, a slice thickness of 1.0 mm and resolution at 0.0078 cm/pixel. T2-weighted MR imaging was obtained using a RARE sequence with the following parameters: RT = 3500.0 ms; TE = 35.22 ms; rare factor = 8, a slice thickness of 1.0 mm and resolution at 0.0078 cm/pixel. MRI scanning was performed 3 weeks post tumour implantation to establish contrast enhancement defining tumour take, and thereafter weekly following start of treatment from day 16 to day 70 during follow-up (Fig. 3a). The same 3 animals per group were followed with Longitudinal MRI scanning, and tumour volumes were calculated in Osirix v 8.5 (Pixmeo, Bernex, Switzerland). Each measurement involved (1) T2-weighted scan and (2) post-contrast T1-weighted scan with 0.1 mL of Dotarem® (Guerbet, Villepinte, France) Tumour volume doubling time was calculated by using the following equation:

$$Doubling time (days) =\frac{duration (days) * log(2)}{\log\left( final tumor volume \right)-\log(intital tumor volume)}$$

*Immunohistochemistry*

Formalin-fixed paraffin-embedded (FFPE) sections of brains were subjected to IHC staining using standard procedures and stained with antibodies indicated in Supplementary Table II. The Ki67 labelling index and CD31 positive microvascular density were quantified in 5 microscopic high-power fields (400× magnification) in all animals in each study group as previously described ^1^.

*Blood, plasma sampling and Platelet counts*

Quantification of platelets in whole blood was performed on fresh blood drawn at the time of sacrifice of animals (n=3, each group) on day 1 and day 2 post BTZ 1.3 mg/m^2^ treatment, with or without tumour bearing control mice or without tumour bearing BTZ 1.3 mg/m^2^ treated mice. The quantification was performed at the department of microbiology and biochemistry clinical laboratory (Haukeland University Hospital, Bergen, Norway) after optimization for mouse values using optical cytometry with the ADVIA® 2120/2120i Hematology System, according to manufacturer’s instructions (Siemens Healthineers; Erlangen, Germany) ^2^.

*Whole blood clotting time*

A whole blood clotting time (WBCT) test was performed on fresh blood drawn at day 1 and day 2 post BTZ 1.3 mg/m^2^ treatment (n=3), compared with control mice (n=3) or without tumour or at day 1 post BTZ 1.3 mg/m^2^ treated mice (n=3) with or without tumour and animals in the other treatment groups (TMZ 82 mg/m^2^ or 164 mg/m^2^ treated and combination BTZ+TMZ treated) (n=6, each group) at the time of sacrifice. The procedure was performed essentially as described previously ^3^. Briefly, blood was drawn into syringe from the opened thorax and placed onto a plastic petri dish. A plastic toothpick was stroked through the blood at 10-sec intervals until the first fibrin stand adhered to the toothpick. WBCT was recorded as the time elapsed from cutting the thorax and blood draw until a fibrin strand was detected.

*Biochemistry analyses of ASAT, ALAT*

500 μl of blood was drawn from the thoracic cavity of animals (n=3, each group) (treatment groups: control mice with or without tumour, BTZ 1.3 mg/m^2^ treated mice with or without tumour, TMZ 82 mg/m^2^ or 164 mg/m^2^ treated mice and combination BTZ+TMZ treated mice) in EDTA coated syringes and thereafter plasma was obtained by centrifugation 2000rpm/20min at 4°C. Alanine aminotransferase (ALAT) and aspartate aminotransferase (ASAT) liver enzymes were also measured in plasma by photometry using the IFCC method at the department of microbiology and biochemistry clinical laboratory (Haukeland University Hospital), where the reaction was initiated by the addition of α-ketoglutarate as a second reagent. The concentration of NADH was measured by its absorbance at 340 nm, and the rate of absorbance was proportional to the ALAT or ASAT activity.

*Sample preparation for proteomics and NanoLC mass spectrometry of tryptic peptides*

Plasma samples were denatured by adding urea solution (8 M urea/20 mM methylamine) before digestion in a solution with modified porcine trypsin (V 5111, Promega). Tryptic peptides (1 µg) were injected into an Ultimate 3000 RSLC system (Thermo Fisher Scientific) connected online with positive electrospray ionization on a LTQ-Orbitrap Velos Pro mass spectrometer (Thermo Fisher Scientific). The tryptic plasma peptides were eluted with a 180 min biphasic acetonitrile (I) gradient from a 50 cm analytical column (Acclaim PepMap100 nanoViper column, 75 μm i.d. × 50 cm, packed with 3 μm C18 beads; Thermo Fisher Scientific) as described ^4^.

*Label-free protein quantification*

The raw MS data were analysed with MaxQuant module version 1.5.2.8 (developed by Computational Systems Biochemistry group, Max Planck Institute of Biochemistry, Martinsried, Germany)using the search engine Andromeda for identification and label-free protein quantification (LFQ). MaxQuant’s settings for LFQ analysis were as previously described ^4^. The mass spectrometry proteomics data and files have been deposited into the ProteomeXchange Consortium via the PRIDE partner repository with the dataset identifier PXD007193.

**Supplementary Table I. Temozolomide and Proteasome inhibitors response to patient derived cells and GBM cell lines**

|  |  | MGMT  Methylation Status | Genetic background | | | | | | Response to Temozolomide  IC_50_ (µM) 72 h | | Response to Proteasome Inhibitor IC_50_ (nM) 48 h | | | |
| --- | --- | --- | --- | --- | --- | --- | --- | --- | --- | --- | --- | --- | --- | --- |
|  |  |  |  |  |  |  |  |  |  |  | Bortezomib | | Carfilzomib | MG-132 |
|  |  |  |  |  |  |  |  |  |  |  | Reversible | | Irreversible | Reversible |
|  |  |  | p53 | EGFR | IDH1 | PTEN | Mdm2 | CDKN2A | Viability | Clonogenic | Viability | Clonogenic | Viability | Viability |
|  | NHA | M | Wt ^5^ | - | - | - | - | - | 2002±28 | 14±2 | 70±3 | 8±1 | 43±10 | 270±24 |
| Patient Derived Cells | P3 | **U** | - | Wt ^6^ | Wt | Mut ^6^ | - | Mut ^6^ | - | 203±5 | 48±15 | 12±1 | 154±43 | 326±23 |
|  | 2012-18 | U/**M** | - | - | Wt | - | - | - | - | 7±1 | 38±7 | 9 | 550±85 | 618±75 |
|  | BG7 | M | - | - | Wt | - | - | - | - | 10±1 | - | 12±1 | - | - |
| GBM Cell lines | TG98 | **U**/M | Mut ^7^ | Wt ^7^ | Wt^8^ | Mut ^7^ | - | Mut ^7^ | 2473±89 | 227±9 | 61±12 | 10±1 | - | - |
|  | U87 | M | Wt  ^9^ | Wt ^7^ | Wt^8^ | Del ex 3 ^9^ | Wt ^9^ | Mut ^9^ | 1363±66 | 27±2 | 190±18 | - | - | - |
|  | HF66 | **U**/M | - | - | - | - | - | - | 1771±40 | 112±6 | - | - | - | - |
|  | A172 | M | Wt ^9^ | Mut ^10^ | Wt^8^ | Del ex 1-10 ^9^ | Wt ^9^ | Mut ^9^ | 1218±36 | 25±3 | - | - | - | - |

NHA= Normal Human Astrocytes, U= Unmethylated and M= Methylated, **-** = Data not Available

**Supplementary Table II: List of antibodies**

All are from rabbit except for anti-IκBα that are from mouse.

| Antibody | Cat. number (Manufacturer) | Dilution |
| --- | --- | --- |
| Rabbit Anti-MGMT | 2739 (Cell Signaling Technology, Danvers, MA, USA) | 1:500 |
| Rabbit Phospho-NF-κB2 p100 (Ser866/870) | 4810T (Cell Signaling Technology) | 1:1000 |
| Rabbit Anti-IKKα | 2862P (Cell Signaling Technology) | 1:1000 |
| Rabbit Phospho-IKKα/β (Ser176/180) | 2697P (Cell Signaling Technology) | 1:1000 |
| Mouse Anti-IκBα | 4814P (Cell Signaling Technology) | 1:1000 |
| Rabbit Phospho-IκBα (Ser32) | 2859P (Cell Signaling Technology) | 1:1000 |
| Rabbit Anti-NF-κB p65 (D14E12) XP® | 8242P (Cell Signaling Technology) | 1:1000 |
| Rabbit Phospho-NF-κB p65 (Ser536) | 3033P (Cell Signaling Technology) | 1:1000 |
| Rabbit Anti-GAPDH  Rabbit Anti-Ki67  Rabbit Anti-CD31 (Pecam-1) | 5174S (Cell Signaling Technology)  M7240 (Dako, CA, USA)  DIA-310 (Dianova, Hamburg, Germany) | 1:1000  1:75  1:20 |
| Goat Anti Rabbit IgG- HRP | 31460 (Thermo Fisher Scientific, Waltham, MA, USA) | 1:10000 |
| Goat Anti Mouse IgG- HRP | sc-2031 (Santa Cruz Biotechnology, Dallas, TX, USA) | 1:10000 |
| Rabbit Anti rat IgG- Biotinylated | BA-4001 (Vector Laboratories, Burlingame, CA,USA) | 1:100 |

5/11 antibodies were not listed in Antibodypedia antibody profile databases. Anti-Ki67 from Dako is a diagnostic grade antibody used in pathology department at Haukeland University Hospital. Multiple methods were used throughout the manuscript to confirm results obtained with immunoblotting or staining.

**References**

1. Wang J, Svendsen A, Kmiecik J, Immervoll H, Skaftnesmo KO, Planaguma J *et al.* Targeting the NG2/CSPG4 proteoglycan retards tumour growth and angiogenesis in preclinical models of GBM and melanoma. *PLoS One* 2011; **6**(7): e23062; doi 10.1371/journal.pone.0023062.

2. Tycko DH, Metz MH, Epstein EA, Grinbaum A. Flow-cytometric light scattering measurement of red blood cell volume and hemoglobin concentration. *Appl Opt* 1985; **24**(9): 1355.

3. Dotson RLL, J.E.; Marengo-Rowe, A.L.; Ulbbelaker, J.E. Hemostatic parameters of the blood of cotton rats, Sigmodon hispidus, infected with parastrongylus costaricensis (Metastrongyloidea: Angiostrongylidae). *Transactions of the American Microscopical Society* 1990; **109**(4): 339-406.

4. Aasebo E, Mjaavatten O, Vaudel M, Farag Y, Selheim F, Berven F *et al.* Freezing effects on the acute myeloid leukemia cell proteome and phosphoproteome revealed using optimal quantitative workflows. *J Proteomics* 2016; **145**: 214-225; doi 10.1016/j.jprot.2016.03.049.

5. Sato Y, Kurose A, Ogawa A, Ogasawara K, Traganos F, Darzynkiewicz Z *et al.* Diversity of DNA damage response of astrocytes and glioblastoma cell lines with various p53 status to treatment with etoposide and temozolomide. *Cancer Biol Ther* 2009; **8**(5): 452-457.

6. Keunen O, Johansson M, Oudin A, Sanzey M, Rahim SA, Fack F *et al.* Anti-VEGF treatment reduces blood supply and increases tumor cell invasion in glioblastoma. *Proc Natl Acad Sci U S A* 2011; **108**(9): 3749-3754; doi 10.1073/pnas.1014480108.

7. Cobanoglu G, Turacli ID, Ozkan AC, Ekmekci A. Flavopiridol's antiproliferative effects in glioblastoma multiforme. *J Cancer Res Ther* 2016; **12**(2): 811-817; doi 10.4103/0973-1482.172132.

8. Ichimura K, Pearson DM, Kocialkowski S, Backlund LM, Chan R, Jones DT *et al.* IDH1 mutations are present in the majority of common adult gliomas but rare in primary glioblastomas. *Neuro Oncol* 2009; **11**(4): 341-347; e-pub ahead of print 2009/05/14; doi 10.1215/15228517-2009-025.

9. Chekenya M, Krakstad C, Svendsen A, Netland IA, Staalesen V, Tysnes BB *et al.* The progenitor cell marker NG2/MPG promotes chemoresistance by activation of integrin-dependent PI3K/Akt signaling. *Oncogene* 2008; **27**(39): 5182-5194; doi 10.1038/onc.2008.157.

10. Fenstermaker RA, Ciesielski MJ. EGFR Intron Recombination in Human Gliomas: Inappropriate Diversion of V(D)J Recombination? *Curr Genomics* 2007; **8**(3): 163-170.

**Figure S1. Normalized platelet counts and clotting time after TMZ treatment.**

Mean ± S.E.M. values from 3-4 animals in the indicated treatment groups (D1: day one, D2: day two, -T: non-tumour bearing animals, and +T: tumour bearing animals) for (A) platelet counts (10^9^/L), (B) whole blood clotting time (sec), (C) alanine aminotransferase (U/L) and (D) aspartate aminotransferase (U/L). (E) LC-MS/MS proteomics data and Perseus generated heat map of proteins that cluster differentially based on frequency and function in the plasma of all animals in the indicated treatment groups. Proteins that are downregulated are indicated in (green), upregulated in (red), unchanged (dark red/brown). Mean ± S.E.M. label free quantification (LFQ) intensity Log2 for (F) proteasome α-4 subunit and proteasome β-1 subunit; and (G) peroxiredoxins -1, -2, -6, thioredoxin and catalase. One-way ANOVA with Bonferroni correction for multiple testing, **P* < 0.05, ***P* < 0.01, ****P* < 0.001 and *****P* < 0.0001.

Figure S2. (A) T1-weighted MRI with contrast of animals (*n* = 3/ group) BG7 tumours treated with 1.3 mg/m^2^ BTZ for one (top panel) vs. two cycles (bottom panel). (B)Longitudinal measurements of animal weight (g) over 34 days. (C) Kaplan-Meier curves showing % survival in days . Longitudinal measurements of animal weight (g) of mice bearing P3 (D) BG7 (E) tumour, **P* < 0.05, ***P* < 0.01.

# Figure S3. Ethidium bromide stained agarose gel showing amplified DNA fragments corresponding to MGMT promoter methylation status in NHA and glioma cells including (HCC1569 breast cancer cells were used as unmethylated control ATCC^®^ CRL-2330^™^). MW: 93 bp, unmethylated; and 81 bp, methylated fragments

#
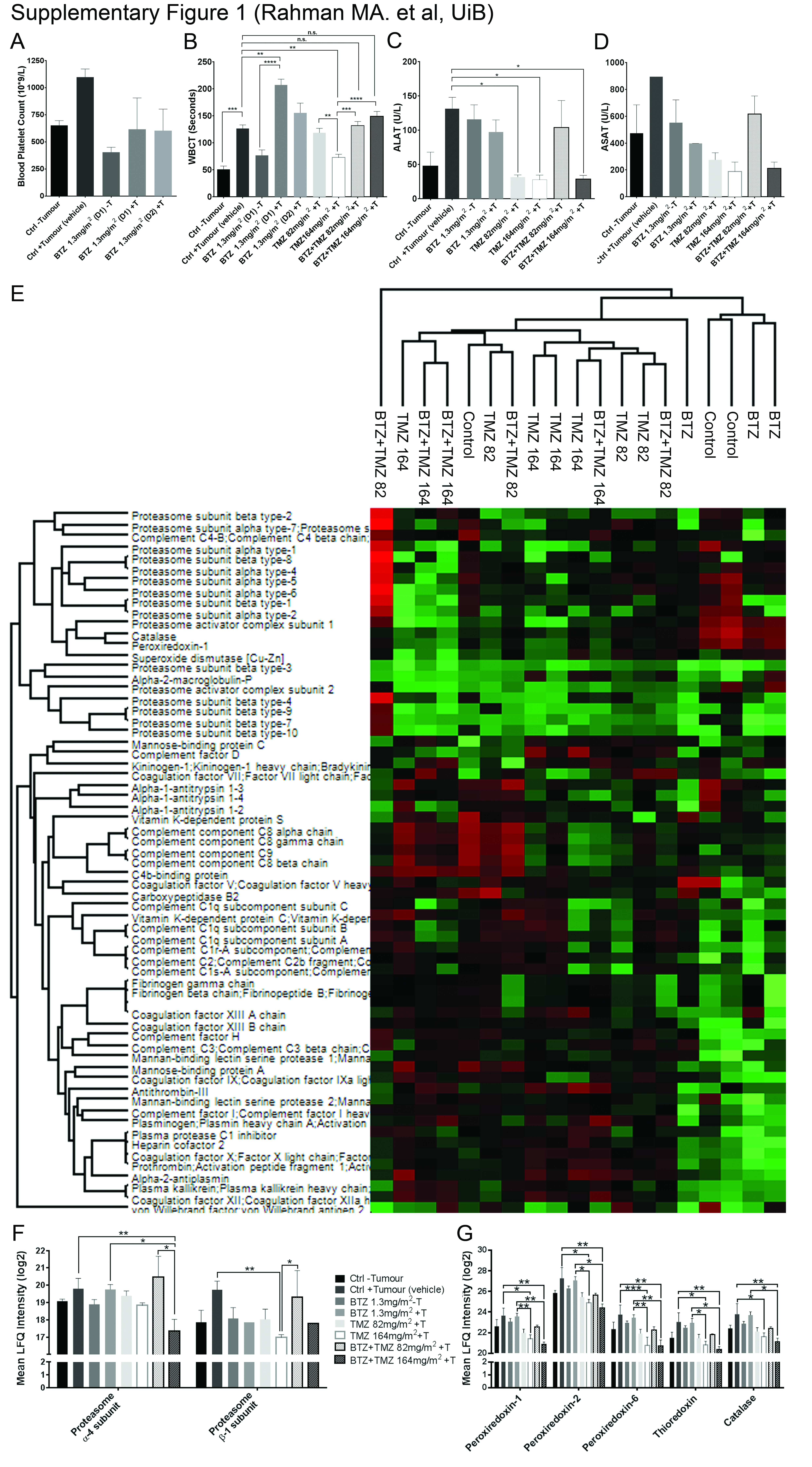


#



#
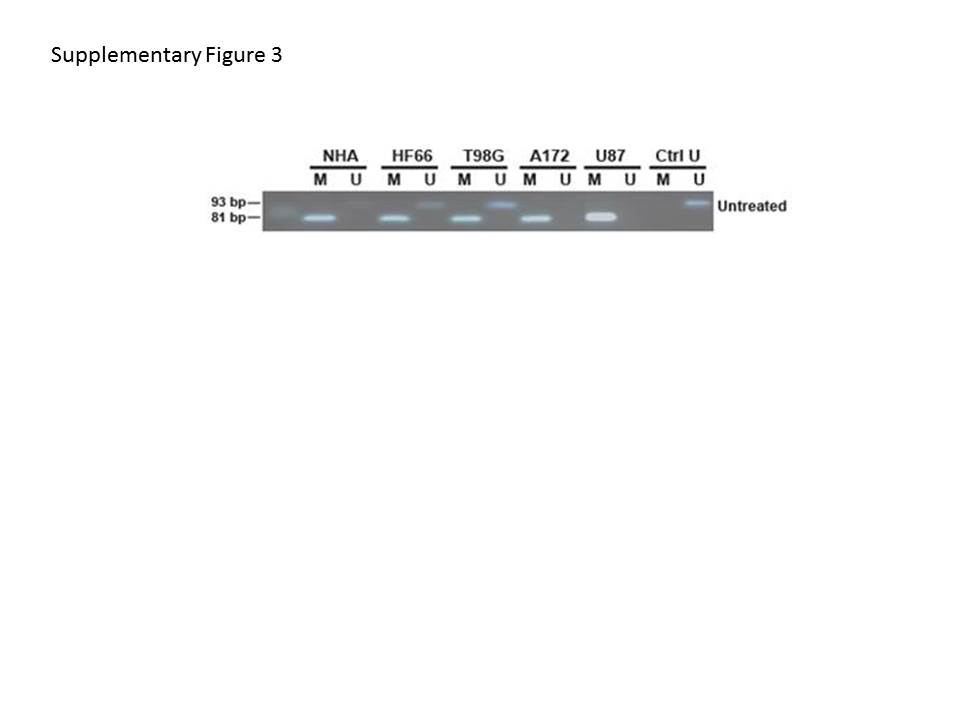

Supplement: Supplementary file 1 — Supplementary Information [file 41416_2019_551_MOESM1_ESM.docx]
